# Selection of O‐negative induced pluripotent stem cell clones for high‐density red blood cell production in a scalable perfusion bioreactor system

**DOI:** 10.1111/cpr.13218

**Published:** 2022-03-15

**Authors:** SuE Yu, Svetlan Vassilev, Zhong Ri Lim, Jaichandran Sivalingam, Alan Tin Lun Lam, Valerie Ho, Laurent Renia, Benoit Malleret, Shaul Reuveny, Steve Kah Weng Oh

**Affiliations:** ^1^ Stem Cell Group Bioprocessing Technology Institute, Agency for Science, Technology and Research Singapore Republic of Singapore; ^2^ Singapore Immunology Network Agency for Science, Technology and Research Singapore Republic of Singapore; ^3^ A*STAR Infectious Diseases Labs Agency for Science, Technology and Research Singapore Republic of Singapore; ^4^ Lee Kong Chian School of Medicine, Nanyang Technological University Singapore Republic of Singapore; ^5^ School of Biological Sciences, Nanyang Technological University Singapore Republic of Singapore; ^6^ Department of Microbiology and Immunology, Immunology Translational Research Programme Yong Loo Lin School of Medicine, Immunology Programme, Life Sciences Institute, National University of Singapore Singapore Republic of Singapore

## Abstract

**Objectives:**

Large‐scale generation of universal red blood cells (RBCs) from O‐negative (O‐ve) human induced pluripotent stem cells (hiPSCs) holds the potential to alleviate worldwide shortages of blood and provide a safe and secure year‐round supply. Mature RBCs and reticulocytes, the immature counterparts of RBCs generated during erythropoiesis, could also find important applications in research, for example in malaria parasite infection studies. However, one major challenge is the lack of a high‐density culture platform for large‐scale generation of RBCs in vitro.

**Materials and Methods:**

We generated 10 O‐ve hiPSC clones and evaluated their potential for mesoderm formation and erythroid differentiation. We then used a perfusion bioreactor system to perform studies with high‐density cultures of erythroblasts in vitro.

**Results:**

Based on their tri‐lineage (and specifically mesoderm) differentiation potential, we isolated six hiPSC clones capable of producing functional erythroblasts. Using the best performing clone, we demonstrated the small‐scale generation of high‐density cultures of erythroblasts in a perfusion bioreactor system. After process optimization, we were able to achieve a peak cell density of 34.7 million cells/ml with 92.2% viability in the stirred bioreactor. The cells expressed high levels of erythroblast markers, showed oxygen carrying capacity, and were able to undergo enucleation.

**Conclusions:**

This study demonstrated a scalable platform for the production of functional RBCs from hiPSCs. The perfusion culture platform we describe here could pave the way for large volume‐controlled bioreactor culture for the industrial generation of high cell density erythroblasts and RBCs.

## Graphical abstract

Human pluripotent stem cell‐derived red blood cells could help alleviate worldwide blood shortages and provide a secure year‐round supply. However, current generation methods lack the necessary scalability. Here, we present the selection of O‐negative hiPSC clones and demonstrate the small‐scale generation of functional erythroblasts in a high‐density perfusion bioreactor system.

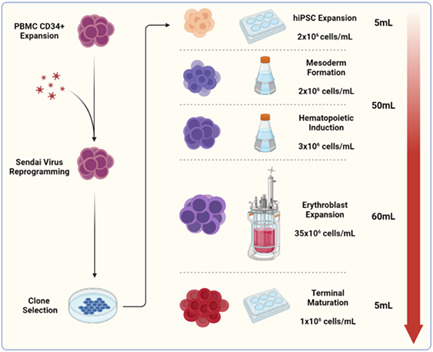

## 1 INTRODUCTION

As populations across the developed world continue to age, the global demand for blood has increased dramatically in recent years.^1^ Collection of blood necessary for transfusions for chronic conditions, incidents and natural calamities has not kept up; in particular, the Covid‐19 pandemic has showcased the difficulty in maintaining a steady year‐round supply. Thus, methods for ex vivo manufacture of red blood cells (RBCs) from human stem cells have been actively explored in recent years. In particular, the generation of O‐negative Rhesus factor D negative (O‐ve) blood cells from human induced pluripotent stem cells (hiPSCs) is widely considered among the most attractive strategies. O‐ve RBCs are the universal donor blood type due to the lack of potentially immunogenic membrane proteins.^2^ Furthermore, hiPSCs have the capacity for virtually unlimited proliferation in vitro and have the potential to differentiate effectively into the haematopoietic lineages.^3^ This makes them an attractive and theoretically limitless source for universal RBC production.

While many groups have reported processes for differentiating hiPSCs towards RBCs, these have not been demonstrated to be scalable.^4^, ^5^, ^6^, ^7^, ^8^ Most have described the use of hiPSCs cultured on 2D planar surfaces, an approach with limited potential for scale‐up due to surface area limitations, increased manpower demand and lot‐to‐lot variability. To overcome this problem, we have developed a continuous suspension agitation culture process for the scalable generation of erythroid cells from hiPSCs using microcarriers. We have previously demonstrated erythroid differentiation of hiPSCs cultured in 125 ml spinner flasks, achieving cell densities exceeding 1.5 × 10^7^ cells/ml.^9^ However, our current platform for RBC differentiation from hiPSCs is still limited by the necessity of achieving high‐density cultures of erythroblasts in order to reduce the use of media and improve downstream processing of the final cell product. Current estimates place the cost of in vitro blood products at US$15,000 or more, mainly due to the multiple stages of differentiation, low cell densities and frequent media changes utilized in most processes described.^10^ Given that each transfusion unit of blood contains 1 × 10^12^ RBCs and that media costs can reach as much as $8000 per unit of hiPSC‐RBCs,^11^ there is a need to reach cell densities greater than 1 × 10^8^ cells/ml in order to achieve high‐density culture in a cost‐effective manner.

Development of a perfusion culture system that allows for continuous media exchange (simultaneous addition of fresh medium and removal of spent medium) while retaining cells in culture could in theory allow for cells to remain in the exponential phase for a prolonged period of time and achieve the desired cell densities. Current perfusion technologies rely on semi‐permeable membranes or hollow fibre cassettes for separation/retention of cells. However, neither of these operates efficiently at high flow rates. Moreover, at high cell densities, there may be issues with clogging of the membrane or hollow fibres which could ultimately affect the quality of the cells.^12^

Applikon® Biotechnology has a patented bioreactor platform, the Applikon BioSep, which uses high frequency resonant ultrasonic waves to separate cells instead of a physical mesh or membrane – a non‐fouling and non‐clogging retention system.^13^ The BioSep system can be mounted directly onto the headplate of a stirred‐tank bioreactor. In addition, the Applikon mini‐bioreactor system (250 ml) allows for the development and testing of small‐scale platforms for optimizing perfusion cultures. Given the high cost of culturing erythroblasts, this represents a cost‐effective approach for process development. A successful perfusion culture platform in the Applikon mini‐bioreactor system can potentially be scaled up to larger controlled bioreactors, thus paving the way for large‐scale manufacturing of high cell density erythroblasts and RBCs. The Applikon BioSep system has previously been used for achieving high density cultures of murine pluripotent cells,^14^ insect cells^15^ and CHO cells.^16^

In this study, we have been able to demonstrate what is to our knowledge the first small‐scale generation of high‐density perfusion cultures of erythroblasts from hiPSCs in a stirred bioreactor system. After process optimization, we were able to achieve a maximum cell density of 34.7 × 10^6^ erythroblasts/ml, which retained a viability of over 90% over the duration of the culture.

## 2 MATERIALS AND METHODS

### 2.1 iPSC generation and culture

Commercial O‐neg peripheral blood mononuclear cell (PBMC) was given as a gift from Dr Jonathan Loh (IMCB, ASTAR). PBMC was first expanded in a 50 ml shake flask after thawing. Four days later, CD34+ cells were isolated using magnetic beads (Miltenyi Biotec) and expanded for a further 3 days. Afterwards, 1 × 10^5^ CD34+ cells were reprogrammed using commercial Sendai virus according to the manufacturer's instructions (Thermo Fisher). Around D14, different colonies were picked and seeded on LN‐521 (BioLamina) coated microcarriers (Solohill® Plastic Plus) for expansion. hiPSCs were cultured on 2D monolayer and 3D microcarriers as described previously.^17^

### 2.2 Tri‐lineage differentiation

Tri‐lineage differentiation was performed for all hiPSC colonies using a STEMdiff™ Trilineage Differentiation Kit according to the manufacturer's instructions (STEMCELL Technologies, Catalogue # 05230).

### 2.3 Erythroid cell differentiation from iPSC in suspension platform

Differentiation of erythroid cells from iPSCs in 3D suspension platform has previously been described.^9^, ^18^ Briefly, hiPSCs were seeded on microcarriers (Solohill® Plastic Plus) for expansion under continuous agitation for 7 days in six‐well ULA plates. HiPSC‐MC aggregates were then subjected to mesoderm formation, haematopoietic specification and erythroid induction. Terminal maturation was induced using primary human WJ1 MSCs or murine OP9 stromal cells as described in.^9^ Absolute number of non‐apoptotic enucleated cells was calculated as follows on each day: (percentage of enucleated cells) × (total number of viable cells).

### 2.4 Perfusion bioreactor culture

Erythroblast cell culture was conducted in a perfusion bioreactor system outfitted with a BioSep ultrasonic filter (Applikon® Biotechnology), along with temperature, dissolved oxygen (DO), agitation, and pH control systems.^15^ One sampling port was installed for sampling. Medium feed and culture harvest were achieved using pumps. The ultrasonic filter was composed of one BioSep ADI 1015 power supply and control system and a resonator assembly.

Bioreactor working volume was 60 ml and operational set points were defined as *T* = 37°C, and DO^14^ = 100% of air saturation (21% of O_2_) or pure oxygen saturation. The bioreactor was inoculated at a density of 6 × 10^6^ cells/ml and cultures were stirred at 70 rpm by an impeller (impeller diameter [Di] = 2.8 cm). pH control was achieved through CO_2_ and alkaline (0.5 M NaOH) addition, while DO was maintained by addition of pure oxygen or air.

A level sensor was used to control the level of culture volume in the bioreactor, with fresh media automatically pumped into the vessel to maintain the defined working volume. Acoustic separation in our perfusion system setup was realized using a 0.3 ml BioSep® ultrasonic chamber (30 mW/ml) fitted to the bioreactor headplate, designed by SonoSep™. A harvest pump was synchronized with the BioSep® ultrasonic chamber for perfusion at the exit port of the resonator chamber to remove spent medium. Fresh medium was pumped in and spent medium removed at controlled rates for perfusion to achieve multiple vessel volume changes per day. Each perfusion cycle ended with a backflush step at a flow rate of 360 ml/h (hold‐up volume of the chamber = 0.7 ml) in order to return cells retained in the acoustic filter back to the bioreactor. Backflushed cells were then resuspended in culture by the impeller.

During perfusion operations, the resonance frequency of the ultrasonic filter was set at 0.3–0.7 MHz. Separation efficiency (SE) of the ultrasonic filter was defined as the percentage of cells retained by the filter and was determined using measurements of cell densities both in the bioreactor (CB) and in the harvest line (CH)^15^ after the ultrasonic filter using the formula: SE = (CB − CH)/CB × 100%. Daily measurements of cell viability and number were performed by nuclei count (NucleoCounter NC‐3000; ChemoMetec), except for enucleation studies, where viability was assessed by Trypan Blue exclusion using a Vi‐CELL Cell Viability Analyser (Beckman Coulter). Supernatant collected throughout the culture process was used to measure the concentrations of glucose, lactate and ammonia using a Bioprofile 100 plus (NOVA). Data is shown for the most successful bioreactor run; each run was performed in parallel with two spinner flasks inoculated using the same cells at the same working volume and cell density.

For further methodological details, please also refer to the Supporting Information S1.

## 3 RESULTS

### 3.1 Generation and characterization of O‐negative iPSC lines

**FIGURE 1:**
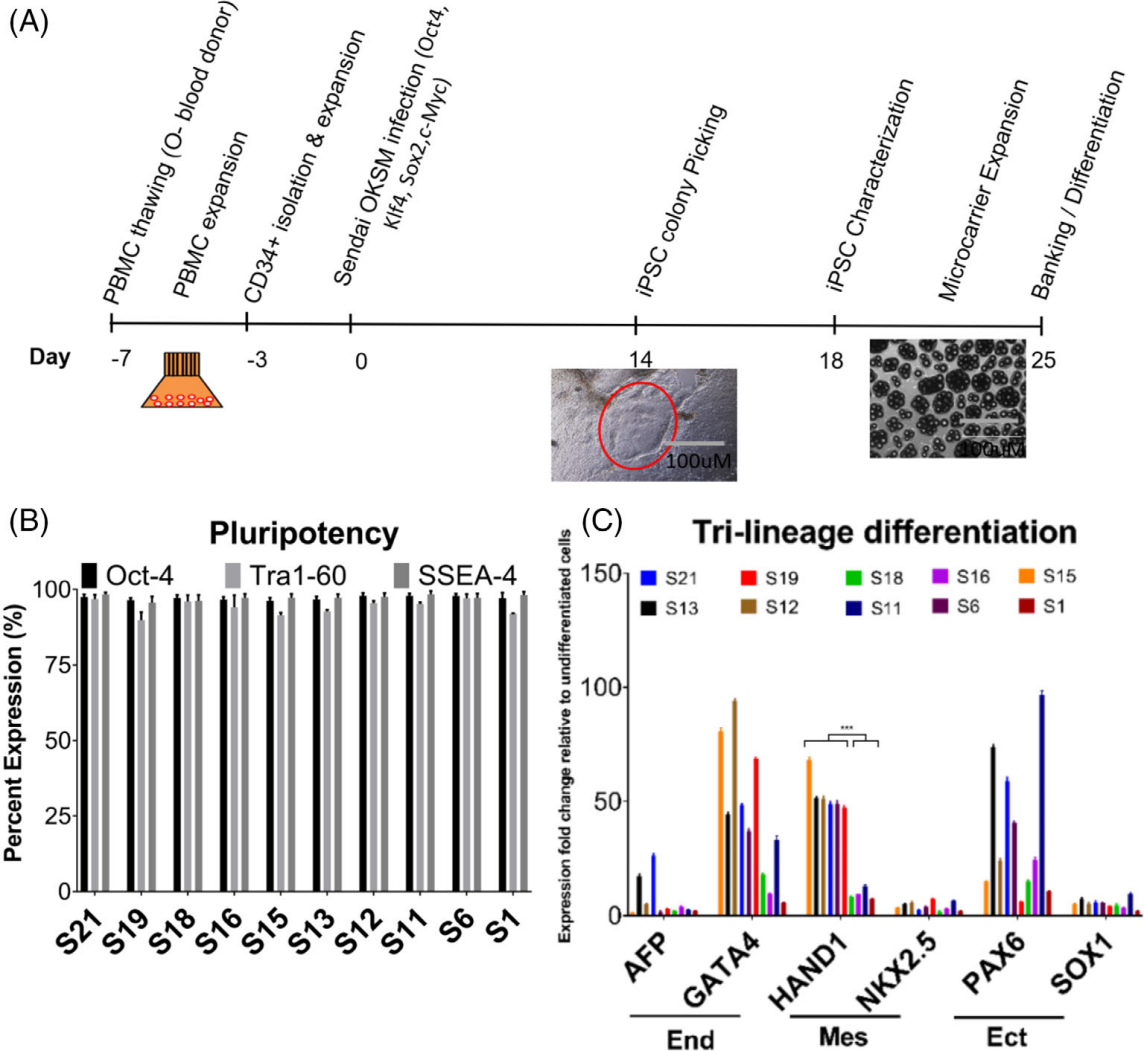
Generation and characterization of different O‐negative iPSC colonies. (A) Workflow of hiPSC generation on the microcarrier platform. (B) Pluripotency marker expression (Oct‐4, Tra1‐60 and SSEA‐4) examined by flow cytometry on Day 18. (C) RT‐qPCR fold‐expression of tri‐lineage markers for 10 iPSC colonies evaluated (Housekeeping gene GAPDH‐normalized) after tri‐lineage differentiation. ***p < 0.001 comparing Hand1 high‐expressing versus low‐expressing group. All data represent mean ± SD with *n* = 3 replicates. Data shown for representative experiment. hiPSCs, human induced pluripotent stem cells; RT‐qPCR, real time quantitative polymerase chain reaction

In order to obtain an appropriate O‐ve hiPSC source for RBC differentiation, we first generated 10 O‐ve hiPSC clones using a microcarrier platform from a commercial PBMC source, which was isolated from a healthy male donor with an O‐negative, Rhesus factor D negative blood type (Figure 1A). PBMCs were expanded in shake flasks and used to isolate CD34+ cells using magnetic beads, which were then expanded for a further 3 days before reprogramming using the Sendai virus method. Around day 14, picked colonies were seeded on recombinant human laminin‐521 (LN‐521) coated microcarriers for expansion. Characterization was then performed using flow cytometry, which showed that all 10 chosen clones expressed high levels of common pluripotency markers (>90% Oct‐4, Tra1‐60 and SSEA‐4) (Figure 1B). To evaluate which clones had the greatest potential for mesoderm formation, we performed a tri‐lineage differentiation experiment as a pre‐screening for the 10 clones, followed by qRT‐PCR to quantify the relative mRNA expression of tri‐lineage markers (AFP/GATA4 for endoderm; PAX6/SOX1 for ectoderm; HAND1/NKX2.5 for mesoderm). Based on the results, six clones (S6, S12, S13, S15, S19 and S21) (48.75–68.1‐fold increase) showed higher relative expression of the mesoderm maker Hand1 compared to the remaining clones (7.5–12.75‐fold increase) (*p* < 0.001) (Figure 1C), indicating these six clones could have greater potential to differentiate towards mesoderm. Additionally, genetic karyotyping for the six selected clones revealed a normal karyotype for each one (Figure S1A).

### 3.2 Evaluation of multiple hiPSC clones for erythroid cell differentiation using a suspension culture platform

Having pre‐screened that the chosen clones (S6, S12, S13, S15, S19 and S21) show greater mesoderm formation potential, we continued with evaluating the differentiation potential of those six iPSC lines towards erythroid cells using our previously described suspension culture platform.^9^

**FIGURE 2:**
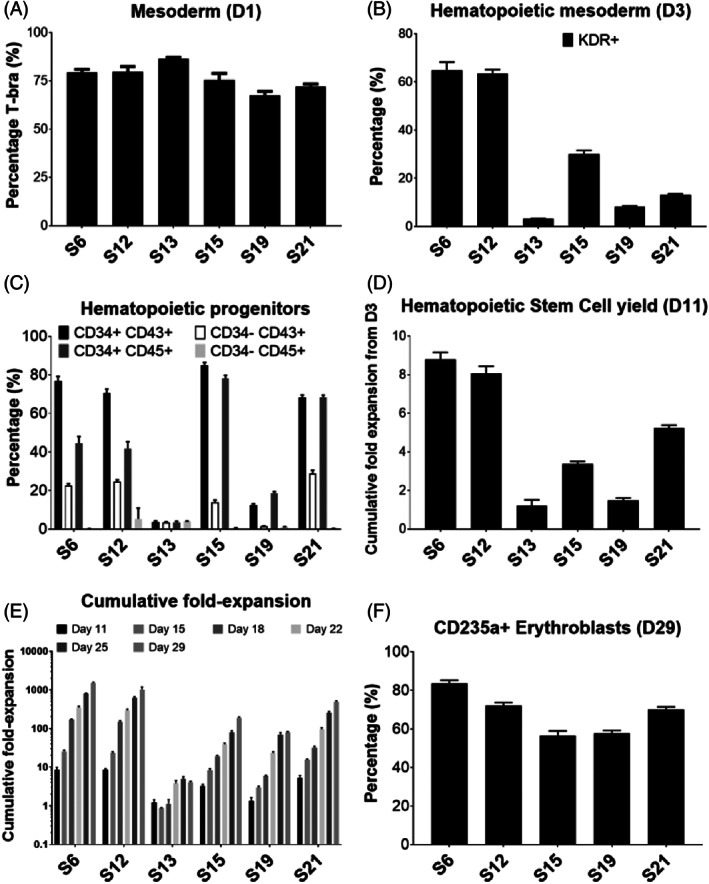
Evaluation of selected human induced pluripotent stem cells colonies for erythroid cell differentiation using the suspension culture platform. (A) Mesoderm/primitive‐streak marker (T‐bra) at differentiation day 1, (B) haematopoietic fated mesoderm markers (KDR+) at differentiation day 3. **p < 0.01 comparing S6 and S12 with other lines. (C) Haematopoietic progenitor markers (CD34+CD43+, CD34+CD45+)/committed haematopoietic cells (CD34‐CD43+, CD34‐CD45+) characterization at day 11. (D) Haematopoietic progenitor cell yield from different clones at day 11 (fold expansion relative to day 3). **p < 0.01 comparing S6 and S12 with other lines. (E) Erythroblast cumulative fold expansion of total viable cells in shake‐flasks from day 11 to day 29. *p < 0.05 comparing S6 with other lines. (F) CD235a characterization of day 29 erythroblasts by flow cytometry. **p < 0.01 comparing S6 and S12 with other lines

iPSC expansion (~7 days prior to differentiation) and mesoderm induction (first 3 days of differentiation) of hiPSC‐microcarrier aggregates were first evaluated in six‐well ULA plates for each clone. Following the 3 days of mesoderm induction, hiPSC‐MC aggregates were trypsinized to obtain single cells, which were then further differentiated in 50 ml shake‐flasks under continuous agitation. All hiPSC clones showed ability to differentiate into T‐Bra+ primitive streak/mesoderm (67.5%–86.4% expression) on day 1 of differentiation (Figure 2A) and showed expression of the haematopoietic fated mesoderm marker KDR+ (3.1%–64.9%) by day 3 (Figure 2B). By day 11 of differentiation, CD34+/CD45+ (3.5%–68.4%) and CD34+/CD43+ (3.5%–84.9%) haematopoietic progenitor cells were detected in all differentiated cultures (Figure 2C). Among the hiPSC clones differentiated, cells derived from S15 had the highest percentage of CD34+/CD43+ (84.9 ± 1.5%) and CD34+/CD45 + (78.15 ± 1.7%) haematopoietic progenitor cells. However, in terms of the cumulative fold growth of haematopoietic progenitor cells for the six clones (which ranged from 1.2 to 8.8‐fold), S6 exhibited the highest yield, showing 8.8 ± 0.36 cumulative fold change relative to day 3 seeding (*p* < 0.01) (Figure 2D). By day 29 of differentiation, S6 also achieved the highest cumulative fold‐expansion of all clones (1510.7 ± 79.8‐fold relative to day 11; *p* < 0.05) (Figure 2E). With the exception of S13, all other five hiPSC clones successfully differentiated into hemoglobinized erythroid cells with CD235a+ expression ranging from 55.9% to 81.8% (Figure 2F; Figure S2A). S6 hiPSC‐derived erythroblasts yielded 83.5 ± 1.7% CD235a+ erythroblasts, once again highest among the six lines differentiated (*p* < 0.05).

Overall, we successfully screened and selected S6 as the best iPSC clone for erythroid cell differentiation, based on its highest production of CD235a+ erythroblasts and overall yield.

### 3.3 High‐density culture of erythroblasts in a stirred bioreactor

**FIGURE 3:**
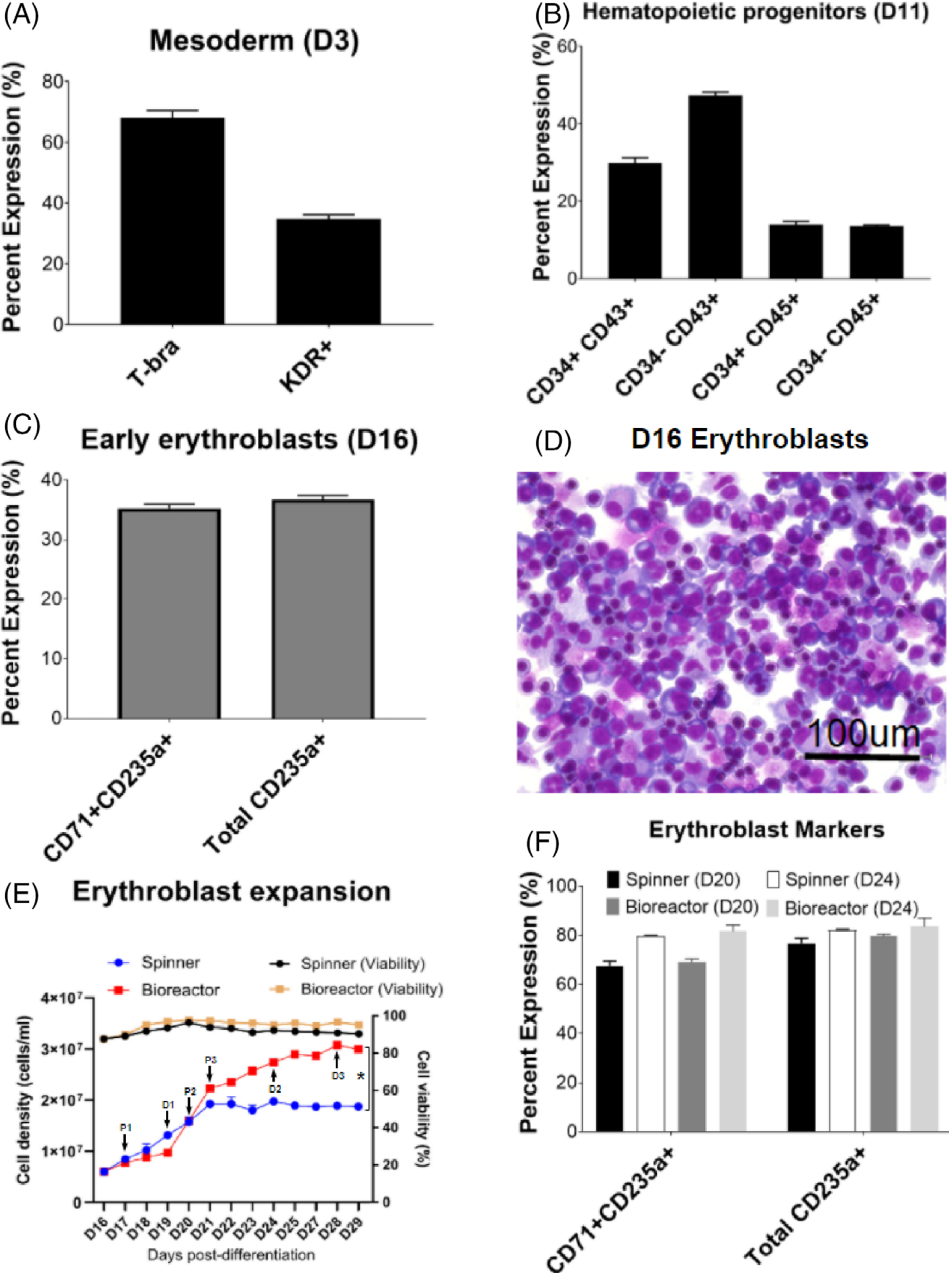
High‐density culture of S6 derived erythroid cells in perfusion bioreactor. (A) Mesoderm/primitive‐streak (T‐bra) and haematopoietic fated mesoderm markers (KDR+) examined at differentiation D3 by flow cytometry. (B) haematopoietic progenitor markers (CD34+CD43+, CD34+CD45+) and committed haematopoietic cells (CD34‐CD43+, CD34‐CD45+) at day 11 examined by flow cytometry. (C) Erythroblasts markers CD235a and CD71 evaluated for day 16 erythroblasts by flow cytometry prior to bioreactor culture. (D) Representative Giemsa staining of S6 derived day 16 erythroblasts. (E) S6‐derived erythroblast expansion and cell viability in perfusion bioreactor and control spinner flask with same culture volume (P1 – perfusion start point; P2/3 – first/second increase in perfusion flow rate; D1/2/3 – first/second/third DO change). *p < 0.05 comparing bioreactor versus spinner growth. (F) Comparison of erythroblast markers CD235a and CD71 for day 20 and day 24 erythroblasts cultured in spinner flasks and bioreactor. Data shown from a highly successful individual bioreactor run & two parallel spinner flasks

After identifying S6 as the best performing hiPSC clone in 50 ml shake flasks for erythroid differentiation, we next attempted to expand S6‐differentiated erythroblasts in a 250 ml (60 ml working volume) stirred‐tank Applikon BioSep perfusion bioreactor system (Figure 3A).

Following S6 iPSC expansion on microcarriers in six‐well ULA plates the cells were subjected to the same differentiation protocol in 50 ml shake flasks as previously performed for haematopoietic induction up until the erythroid expansion stage. Differentiation of S6 was once again assessed using the markers previously described; expression of the primitive streak/early mesoderm marker, T‐bra (68 ± 2.4%), and the haematopoietic fated mesoderm marker, KDR+ (34.7 ± 1.4%), was detected on day 1 and 3 post differentiation, respectively (Figure 3A; Figure S6). CD34+CD43+ (29.8 ± 1.3%) and CD34+CD45+ (13.9 ± 0.8%) haematopoietic progenitors as well as CD34‐CD43+ (47.3 ± 0.9%) and CD34‐CD45+ (13.6 ± 0.26%) committed haematopoietic cells were also detected at day 11 post differentiation (Figure 3B).

At day 16 post differentiation, erythroid cells with early erythroblast marker CD71+/CD235a+ (35.28 ± 0.54%) (Figure 3C,D; Figure S7A) expression were inoculated into the bioreactor at 60 ml working volume at a density of 6 million cells/ml and 87.5% cell viability. DO was set at 10% using pure oxygen supply after calibration and agitation speed was maintained at 70 rpm.^4^ pH control was not set but maintained between pH 7.2 and 7.4. As a control for the bioreactor, erythroid cells with the same density and culture volume were expanded in two 125 ml spinner flasks from day 16 onwards. Perfusion was started at day 17 post differentiation with 60 ml media exchanged per day initially in the bioreactor, while the same volume of fresh medium was changed daily for the spinner flask cultures.

Based on the results shown in Figure 3E, from day 16 to day 19, both cell density and viability in the two conditions increased progressively. However, it was noted that the spinner flask erythroblasts grew faster than those in the bioreactor. In order to investigate the reason for the relatively lower growth rate in the bioreactor, we first examined the SE of the ultrasonic chamber, which showed greater than 99% SE from day 17 to day 19. This, therefore, suggested that the problem did not stem from cell losses incurred due to the perfusion system itself.

#### 3.3.1 Maintenance of erythroid cell growth in bioreactor

Next, metabolite concentrations in the waste medium were assessed. Even though the lactate concentration was slightly higher than the reported inhibitory level of >1.36 g/L, both conditions showed similar concentrations of lactate and ammonia (inhibitory level >4 mM)^19^ (Figure S3A,B), leading to the conclusion that inhibitory metabolites were not the reason for the growth rate difference observed. Given that pH and agitation rate were similar between the two conditions, we theorized that the DO concentration may explain the inhibited growth. At day 19 the DO setting was changed from 10% to 5%, following which a dramatic increase in cell proliferation was observed from day 19 to Day 21. At day 21, the cell density and viability in the bioreactor (22.3 million cells/ml with 97.3% viability) were higher than those observed in the spinner flask (19.2 million cells/ml with 93.8% viability). However, the singular nature of the bioreactor run is insufficient to determine whether these observations were correlated with the DO modulation or simply batch variability. At day 20, we detected that the lactate concentration in the waste medium from the bioreactor was above the inhibitory level of 1.36 g/L^19^ (Figure S3A) and the perfusion rate was increased to 80 ml/day. From day 21 onwards, we observed a slower growth rate in the bioreactor relative to the period from day 19 to day 21. Increasing the perfusion rate to 120 ml/day failed to restore the higher growth rate; cell proliferation between days 23 and 24 remained similar to that observed between days 21 and 23. Furthermore, a decline in cell viability was also noted, from 97.3% (day 21) to 95% (day 24), leading to a speculation that oxygen supply was insufficient to maintain the large number of cells. Therefore, DO was recalibrated from 5% to 8% at day 24. An increase in cell viability was observed at day 25, but the cell growth rate remained similar to that calculated for the period from day 21 to day 24. From day 25 to day 29, the cell density stagnated around 30 million cells/ml, despite an additional increase in DO to 10% at day 28. Overall, we achieved a peak density of 30.8 million erythroblasts/ml with 96.6% viability. In contrast, the control spinner flask cultures were only able to achieve a peak density of 20.2 million cells/ml, with little change between days 21 and 29. In addition, spinner cell viability dropped continuously from 96.2% (day 20) to 90.2% (day 29).

#### 3.3.2 Observations on the effects of pH and oxygen supply on bioreactor cultures

Bayley et al previously reported that pH 7.4 resulted in the highest enucleation efficiency, compared to pH of 7.3 or 7.5.^19^ To test this, pH control was set to 7.4 immediately after inoculation. However, erythroblast growth rates in the pH‐controlled bioreactor were severely inhibited relative to the unoptimized spinner flask cultures. Therefore, in order to mimic the spinner flask environment in the bioreactor, we attempted to repeat the process without controlling pH for the initial days following inoculation. This resulted in a comparable growth rate in both conditions (data not shown). Therefore, we opted to omit pH control in the bioreactor.

Finally, we also conducted a bioreactor run by supplying the culture with air versus pure oxygen. DO was set at 10% (equivalent to 2% DO with pure oxygen supply), and a higher peak density 34.7 million cells/ml with 92% cell viability was achieved (Figure S2C) compared to the Figure 3E result. A possible hypothesis for obtaining a higher density could be that the relatively lower air supply may have a stimulatory effect on cell growth, consistent with the result shown in Figure 3E (5% DO from day 19 to day 21 and 10% DO from day 16 to day 19). Further studies are warranted to fully explore the effect of DO on cell proliferation.

#### 3.3.3 Marker expression comparison between bioreactor and spinner cultures

In order to determine the difference in CD235a expression in the two conditions, we performed flow cytometry with erythroblast markers for day 20 and day 24 erythroblasts from both spinner flask and bioreactor. This revealed no statistically significant difference in total CD235a+ and CD235+/CD71+ expression between the two conditions (Figure 3F; Figure S7B,C). Additionally, virtually all CD235a+ expressing cells in both the bioreactor (81.82 ± 2.5%) and the spinner flask (79.64 ± 0.14%) were late‐stage CD235a+/CD71+ erythroblasts (82.2%–83.76%). This indicates that the bioreactor stirring and perfusion operations, while possibly impacting cell growth and proliferation, did not seem to have a detrimental effect on maturation and CD235a expression.

### 3.4 Efficient terminal maturation of erythroblasts with a shorter process

Terminal maturation of erythroblasts is the critical final step for the efficient production of functional RBCs. However, hiPSC‐derived erythroid cells are associated with low enucleation efficiency, a major bottleneck for large‐scale manufacturing. Our group has previously shown that co‐culture of hiPSC‐derived erythroblasts with the murine stromal cell line OP9 results in a substantial increase in enucleation.^9^

**FIGURE 4:**
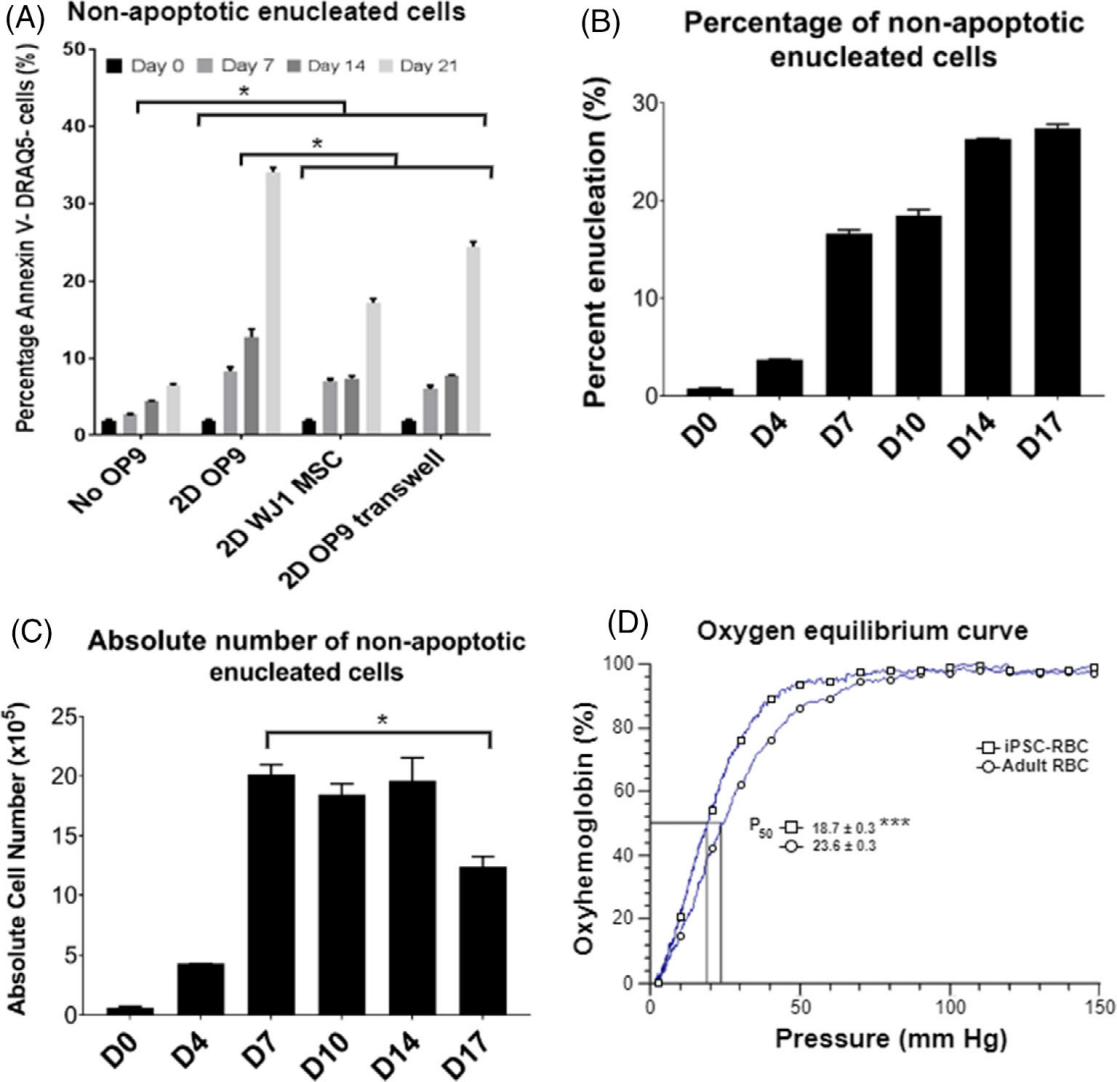
Terminal maturation of erythroblasts. (A) Flow cytometry evaluation for non‐apoptotic (Annexin V‐) enucleated RBCs (DRAQ5‐) of hiPSC erythroblasts after 21 days of terminal maturation without OP9 co‐culture (No OP9), with monolayer OP9 co‐culture (2D OP9), with monolayer bone marrow WJ1 MSC co‐culture (2D WJ1 MSC) and monolayer OP9 co‐culture with transwell plates (2D OP9 transwell). *p < 0.05 comparing No OP9 with all others and 2D OP9 with 2D WJ1 and 2D transwell. (B) Flow cytometry evaluation and (C) absolute number of non‐apoptotic (Annexin V‐) enucleated RBCs (DRAQ5‐) of S6 derived erythroblasts over the course of 17 days of terminal maturation with monolayer OP9 co‐culture. *p < 0.05 comparing enucleation at day 7 and day 17. (D) Oxygen equilibrium curves (percentage oxyhemoglobin versus oxygen pressure in mm Hg) for adult RBCs and S6 erythroblasts. ***p < 0.001 comparing P_50_ for adult vs iPSC‐RBCs. All data represent mean ± SD with *n* = 3 replicates

In order to investigate the reason for this improved enucleation, we performed a 3‐week maturation experiment with OP9 and human mesenchymal line WJ1 cocultures. The results showed that enucleation efficiency (as measured by the DRAQ5 negative, Annexin V negative [i.e., non‐nucleated, non‐apoptotic] population) was highest with OP9 co‐culture (30%), followed by OP9 transwell culture (25%), WJ1 co‐culture (18%) and finally culture without OP9 (8%) (*p* < 0.05) (Figure 4A).

In previous studies, several groups performed erythroblast maturation for 2–3 weeks.^20^, ^21^, ^22^ However, over the course of the 3‐week maturation, we observed that with every medium change, the recovered cell pellet was becoming noticeably smaller, indicating a possible decrease in total cell number. Therefore, in order to investigate the absolute number of enucleated cells over the course of the maturation process, we performed an additional maturation experiment with OP9 coculture. Flow cytometry at days 0, 4, 7, 10, 14 and 17 was performed to quantify the percentage of enucleated cells (Figure 4B), while numbers of enucleated cells were also calculated based on the total number of cells in the culture (via Trypan Blue exclusion) and the enucleation percentage (Figure 4C; Figures S4 and S5). Surprisingly, despite observing a progressive increase in the percentage of enucleated cells over the course of the experiment, the absolute number of enucleated cells remained stagnant and significantly dwindled at 17 days (*p* = 0.02), despite an increase in the percentage of enucleated cells. Peak enucleation (2 × 10^6^ cells) thus occurred on day 7 based on absolute numbers. This result indicates that it is possible to shorten the maturation stage from 21 to 7 days, which would reduce both time and media consumption significantly.

Finally, we generated oxygen equilibrium profiles of the best performing cell line, S6, and compared its oxygen binding affinity to that of adult peripheral blood RBCs. Equilibration curves (Figure 4D) of S6 erythroblasts showed a significant left‐shift (*p* < 0.001), suggesting a higher expression of foetal haemoglobin, which has a higher affinity for oxygen. This is consistent with previous studies by our group, which have shown that erythroblasts obtained using this method have high foetal haemoglobin expression.^9^, ^17^

## 4 DISCUSSION

Evaluation of various starting material sources for in vitro RBC production (e.g., cord blood HSCs, embryonic stem cells and iPSCs) has been widely investigated in recent years. In particular, differentiation of human iPSCs towards the erythroid lineage has been hailed as one of the most attractive approaches, because of its unique capacity for unlimited in vitro proliferation.^4^, ^5^, ^6^, ^8^ However, these methods present challenges pertaining to their scalability, having been performed in either non‐scalable 2D systems or in small‐scale culture unsuitable for robust RBC generation at the industrial scale.

Having noted that each transfusion unit of blood contains 1 × 10^12^ RBCs, it is of vital importance to develop a platform that is capable of achieving high‐density culture for RBCs in a cost‐effective manner. In recent years, several attempts have been made for high‐density manufacture of RBCs in scalable bioreactors.^19^, ^23^, ^24^ For instance, Timmins et al successfully developed a robust high‐yield RBC expansion approach using fully defined culture medium, potentially capable of generating more than 500 units of RBCs per unit of donated umbilical cord blood, while Bayley et al demonstrated the differentiation of cord blood derived CD34+ cells to RBCs in a ml‐scale Ambr™ stirred tank bioreactor system. More recently, Heshusius and colleagues achieved a 3 × 10^7^ – fold erythroblast expansion from PBMC using a G‐Rex bioreactor and GMP‐grade medium over 25 days. However, these studies are related to RBCs differentiated from adult blood or cord blood, but not iPSC, and thus carry some of the same issues as donor blood, such as an irregular and difficult supply of starting material. To date, no groups have been able to demonstrate generation of high‐density cultures of hiPSC‐RBCs in a bioreactor.

Prior to this study, our group established a suspension culture platform for RBC differentiation from hiPSC.^9^, ^17^ Utilizing this platform, we have successfully achieved (to our knowledge) the highest density culture of human iPS‐derived erythroblasts (34.7 million cells/ml) in a perfusion bioreactor setting, with over 90% viability throughout the culture period (Figure S3C).

There are several key advantages of the Applikon® Biotechnology perfusion bioreactor approach utilized in this study. First, the BioSep separation system is a non‐fouling and non‐clogging retention device that eschews the use of a physical mesh or membrane and instead relies on high frequency resonant ultrasonic waves to achieve cell separation. Indeed, no clogging or fouling of the device was observed even at cell densities of over 30 million cells/ml. Second, the system has very robust SE of more than 99%, significantly minimizing cell loss over the culture duration. Finally, BioSep separation is in theory applicable at scales ranging from R&D (perfusion rate of 60 ml/day up to 1 L/day), to process development (up to 10–50 L/day) and industrial manufacturing (perfusion rate of up to1000 L/day). The R&D scale investigated here could serve as the basis for pilot scale studies for RBC manufacturing.

One clear limitation of this experimental setup is that, with just a single vessel, it is not possible to compare several conditions/parameters in parallel (e.g., pH, DO, agitation speed). This could be remedied in future studies using multiple parallel mini bioreactors. Although the high peak density of 35 million cells/ml in this study is a vast improvement over traditional 2D static models, further optimizations of key bioreactor and culture parameters are still warranted. It is likely that the highest density erythroblast culture is yet to be achieved. Furthermore, we noted major differences in both differentiation and proliferative capacity between our hiPSC clones, which leads us to hypothesize that a better differentiating cell line may further increase yields.

Last, overcoming the challenge of low enucleation efficiencies of hiPSC‐derived erythroblasts is vital towards the potential clinical applications of in vitro RBCs. Several research groups have demonstrated that co‐culture of erythroid cells with OP9 or other stromal cells could be helpful to prevent erythroblast apoptosis and promote enucleation.^20^, ^21^, ^22^, ^25^, ^26^ This, however, introduces another obstacle to the scalability of the system. The results from our maturation experiment performed in transwell plates (Figure 4A) suggest that the improved enucleation observed with OP9 co‐culture could be a combination of paracrine effects from proteins secreted by the OP9 cells, as well as cell‐cell interactions between OP9 cells and erythroblasts. Future work could focus on identifying secreted and surface proteins capable of stimulating erythroblasts, paving the way for the development of a defined conditioned enucleation medium without co‐culture.

In conclusion, we have established a perfusion bioreactor system capable of achieving high‐density culture of hiPSC‐derived erythroblasts with a highly efficient cell separation system for a peak density of 34.7 million cells/ml. We show high levels of expression of mature erythroid cell markers and demonstrate that the cells maintain a high viability of over 92% throughout the culture period. We also demonstrate a 7‐day enucleation of RBCs with oxygen carrying capacity. This platform maximizes cell proliferation potential and provides a stepping stone towards large‐scale controlled bioreactors production in the future.

## CONFLICT OF INTEREST

Dr. Steve Oh has patents on microcarrier technology for stem cell cultivation filed by A*STAR. He is also a founder of Zenzic Labs and SingCell. The rest of the authors declare no competing interests.

## AUTHOR CONTRIBUTIONS

Steve Kah Weng Oh was the principal investigator and takes primary responsibility for the article. Yu SuE, Svetlan Vassilev, Zhong Ri Lim, Jaichandran Sivalingam, Alan Tin Lun Lam and Valerie Ho contributed towards the laboratory work. Yu SuE, Svetlan Vassilev, Benoit Malleret, Laurent Renia, Shaul Reuveny and Steve Kah Weng Oh wrote the manuscript.

## Supporting information


**Appendix S1**: Supporting Information

## Data Availability

Data are available from the corresponding author upon reasonable request.
