# Supplementary material for: Selection of O‐negative induced pluripotent stem cell clones for high‐density red blood cell production in a scalable perfusion bioreactor system
Source: Cell Prolif. 2022 Mar 15;55(8):e13218. doi: 10.1111/cpr.13218 (PMC9357363; doi:10.1111/cpr.13218)
Supplement: Supplementary file 1 — Appendix S1: Supporting Information [file CPR-55-e13218-s001.docx]

**Supplemental Materials and Methods**

***RNA extraction and quantitative real-time polymerase chain reaction***

Cell samples were lysed in Trizol® reagent (ThermoFisher Scientific), followed by RNA extraction using Direct-zol™ RNA extraction kit (Zymo Research) according to the manufacturer’s instructions.

After quantifying RNA concentration for extracted RNA samples by OD260 nm measurements, 500 nanograms of total RNA was used for first strand cDNA synthesis using iScriptTM Advanced cDNA synthesis kit (BioRad). cDNA samples diluted 1:10 in RNAse-free water were used for quantitative real-time polymerase chain reaction (PCR) using gene-specific primers (Table S1), iTAQ™ Universal SYBR® green supermix (BioRad), and Applied Biosystems 7500 FAST Real-time PCR system (ThermoFisher Scientific). GAPDH was used as a house-keeping gene for normalization of sample quantities. Relative change in gene expression was determined using the delta-delta c(t) method.

***Flow cytometry***

Flow cytometry was performed as previously detailed [1] on cells fixed with 4% paraformaldehyde (eBioscience) using a NovoCyte Flow cytometer (ACEA Biosciences Inc., USA) and analyzed using FlowJo Software. The following antibodies were used for measuring: pluripotency [primary antibodies: 1:100 Oct4 (R&D Systems, USA); 1:50 Tra1-60 (Millipore), 1:100 SSEA4 (BioLegend, USA) and secondary antibody: 1:500 diluted rabbit anti-mouse IgG-FITC conjugate (DAKO)], mesoderm [T-brachyury-FITC (R&D Systems), KDR-PE (Miltenyi Biotec), hematopoietic marker [CD34-APC, CD43-FITC, CD 45-PE, CD71-APC, CD235a-FITC (all from BD Biosciences, USA)]. The following antibodies were used as isotype-controls: mouse IgG1-FITC and PE (Miltenyi Biotec, Germany), mouse IgG2b_k_-FITC and mouse IgG2a_k_-APC (BD Biosciences). For hemoglobin analysis, 0.1% v/v Triton X-100 permeabilized cells were incubated with 1:50 diluted fetal hemoglobin-FITC (ThermoFisher Scientific) or adult hemoglobin-PE antibodies (Santa Cruz Biotechnology, USA). Detection of enucleated cells was

performed using cells stained with 1:100 diluted CD235a-FITC, 1:100 diluted Annexin V and 1:5000 dilution of a cell-permeable nuclear dye, DRAQ-5™ (eBioscience).

***Immunohistochemistry and microscope imaging***

Cells were cytospun onto glass microscope slides (Marienfled) using Cytospin™ 4 cytocentrifuge (Thermofisher Scientific), fixed and stained using Giemsa stain (Sigma-Aldrich) as detailed previously. Slides were imaged using Axiovert 200M inverted microscope (Zeiss). Immunofluorescence imaging of terminally matured erythroblasts was done using Nikon Eclipse Ti-E fluorescence microscope (Nikon).

***Oxygen equilibration curve***
A Hemox analyzer model B (TCS Scientific Corp) was used to generate the oxygen binding and dissociation equilibration curves of cells as detailed previously [1]. Adult peripheral blood (donor derived) was run as control. All samples were measured in triplicates.

***Statistical analysis***

The statistical analysis was performed either by student’s unpaired *t* -test (comparison for 2 groups) or by one-way ANOVA (comparison for more than 2 groups) using GraphPad Prism 8 (GraphPad Software Inc.) software. Data are presented as mean ± SD. P values < 0.05 were considered statistically significant for *p < 0.05, **p < 0.01, ***p < 0.001.

**Table S1.** List of genes and primers used in PCR studies

| Category | Transcript | Forward Primer (5’-3’) | Reverse Primer (3’-5’) |
| --- | --- | --- | --- |
| Endoderm | AFP | GGGAGCGGCTGACATTATT | GAGCTTGGCACAGATCCTTAT |
|  | GATA4 | GGAAGCCCAAGAACCTGAATA | ACATCGCACTGACTGAGAAC |
| Mesoderm | Hand1 | GCTCTCCAAGATCAAGACTCTG | CCTTTAATCCTCTTCTCGACTGG |
|  | NKX2.5 | AAGTGTGCGTCTGCCTTT | TTTCGGCTCTAGGGTCCTT |
| Ectoderm | PAX6 | AATTGAGGCCCTGGAGAAAG | GGTGCTGAAACTACTGCTGATA |
|  | Sox1 | CAGTACAGCCCCATCTCCAAC | GCGGGCAAGTACATGCTGA |
| Housekeeping | GAPDH | GTCAACGGATTT GGT CGTATT | TGGAAGATGGTG ATGGGATTT |

**Figure S1. Genetic karyotyping for different clones.**A) Genetic karyotyping performed for different hiPSC clones. iPSC culture supernatant from different colonies was collected and high-resolution digital karyotyping (iCS-digital Pluri test) was performed in Stem Genomics.

**Figure S2.**

A) Representative Giemsa staining of D29 erythroblasts derived from different clones.

**Figure S3. Comparison of erythroblast high-density culture in spinner and bioreactor**

Measurements of A) lactate (g/L) and B) ammonia from cell culture supernatants of S6 derived erythroid cells during the erythroblast expansion period in bioreactor and spinner. C) S6-derived erythroblast expansion and cell viability in perfusion bioreactor and control spinner flask with same working volume. 10% DO with air supply was used in this batch. Data shown from a highly successful individual bioreactor run & 2 parallel spinner flasks.

**Figure S4.**

1. Representative DRAQ5-AnnexinV enucleation staining, with D0 being the start of the maturation process with OP9 coculture. The red circles indicate the double negative enucleated population.
2. Representative image of fresh/filtered enucleated erythroblasts following maturation.

**Figure S5.** Representative Giemsa stain image of enucleated erythroblasts during maturation experiment.

1. Day 4 post-start of OP9 maturation (4.9% enucleated)
2. Day 7 post-start of OP9 maturation (15.6% enucleated)

**Figure S6.** Representative flow cytometry gating of the mesoderm and hematopoietic stages of the iPSC-RBC process.

1. D1 T-Bra mesoderm marker.
2. D3 KDR+PDGFRa- hematopoietic mesoderm marker.
3. D11 CD34 & CD43 and CD34 & CD45 hematopoietic stem cell markers.

**Figure S7. Flow plots for CD235a+CD71+ stained erythroblasts at various stages of differentiation**

1. D16
2. D20
3. D24


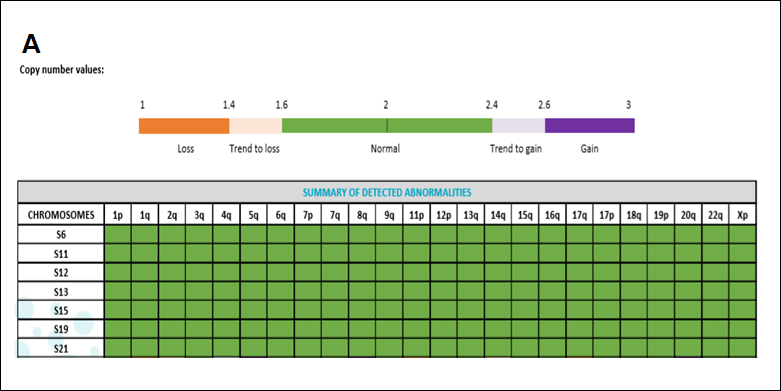


**Figure S1.**


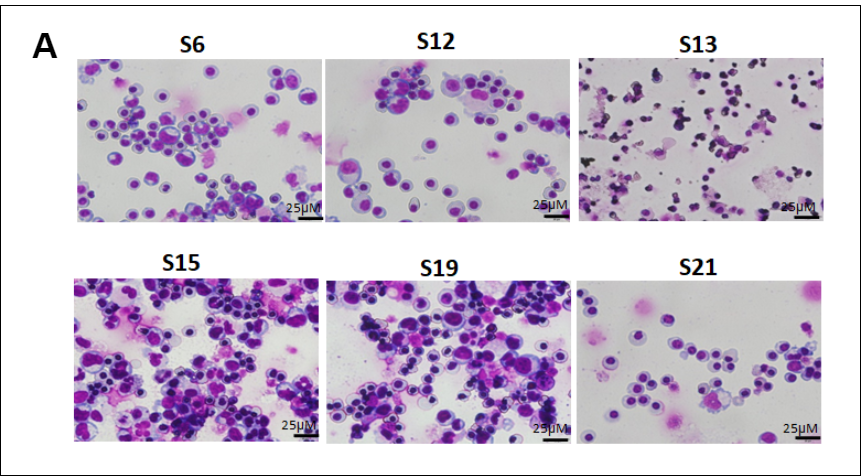


**Figure S2.**


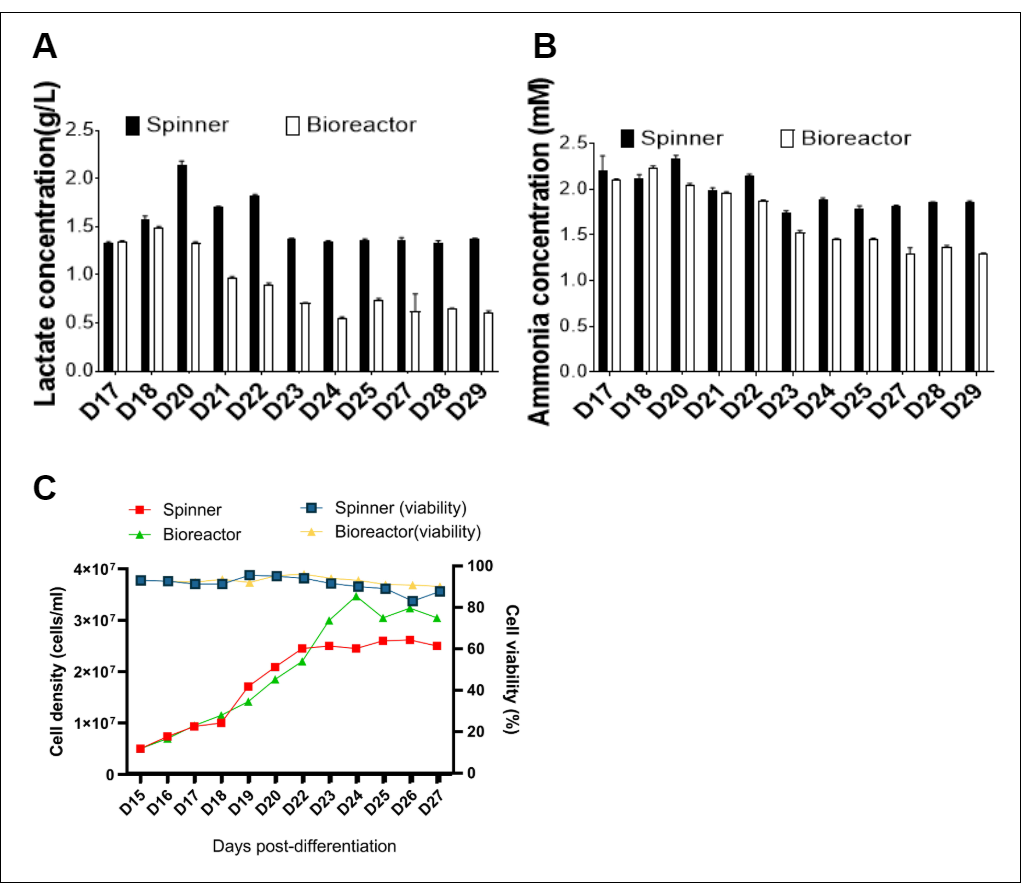


**Figure S3.**


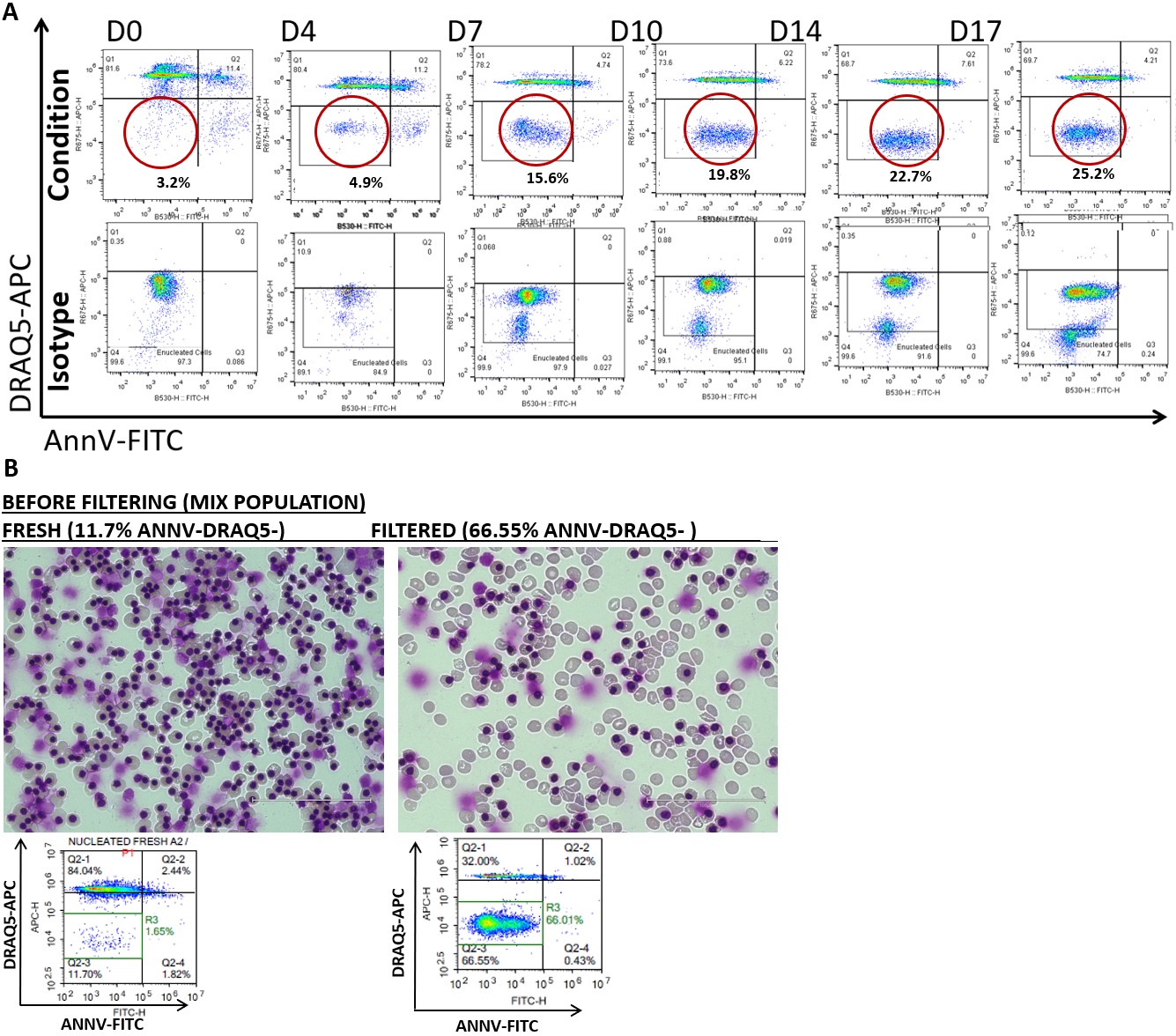


**Figure S4.**


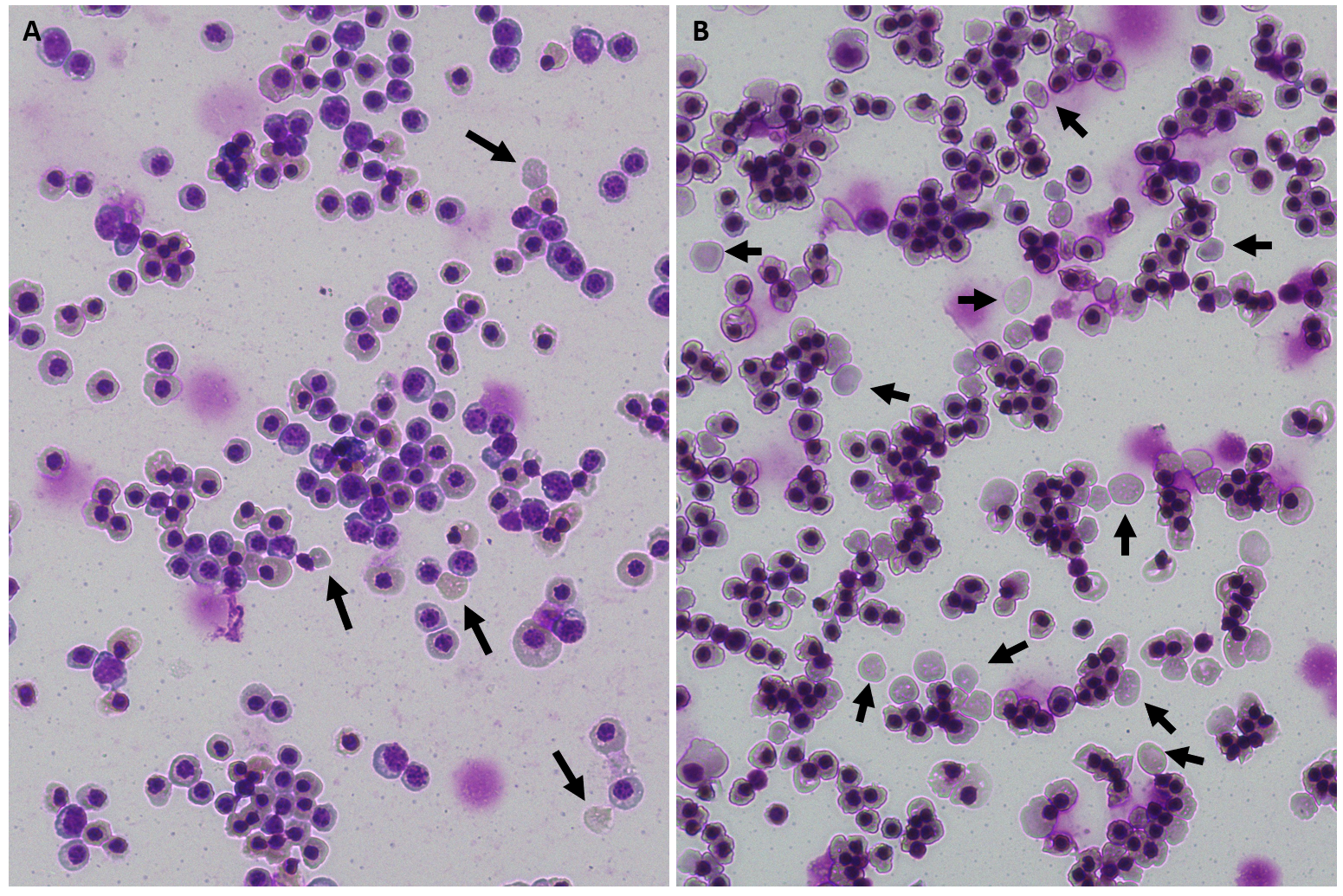


**Figure S5.**


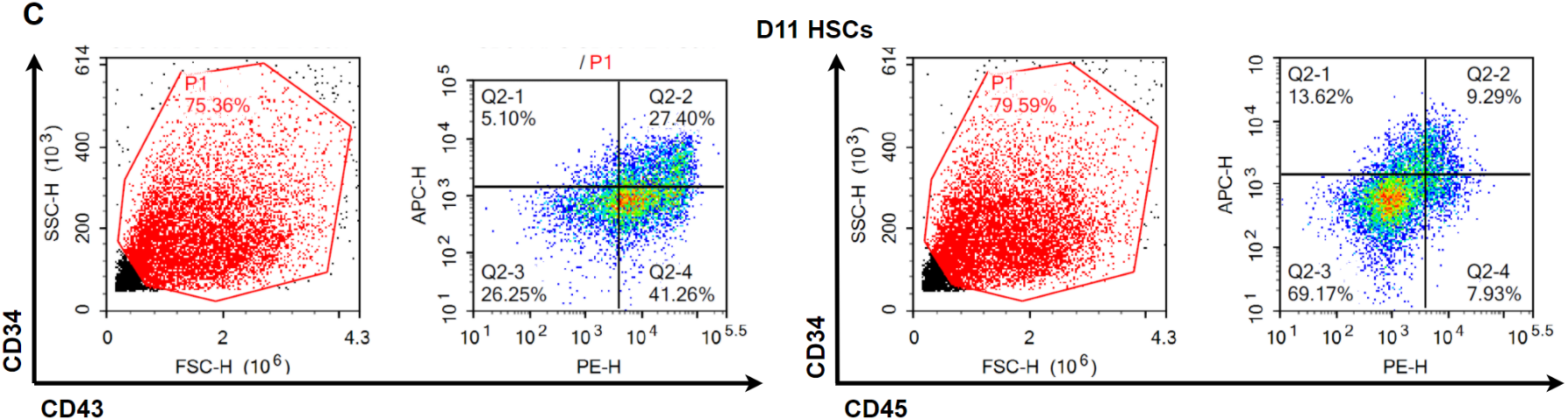

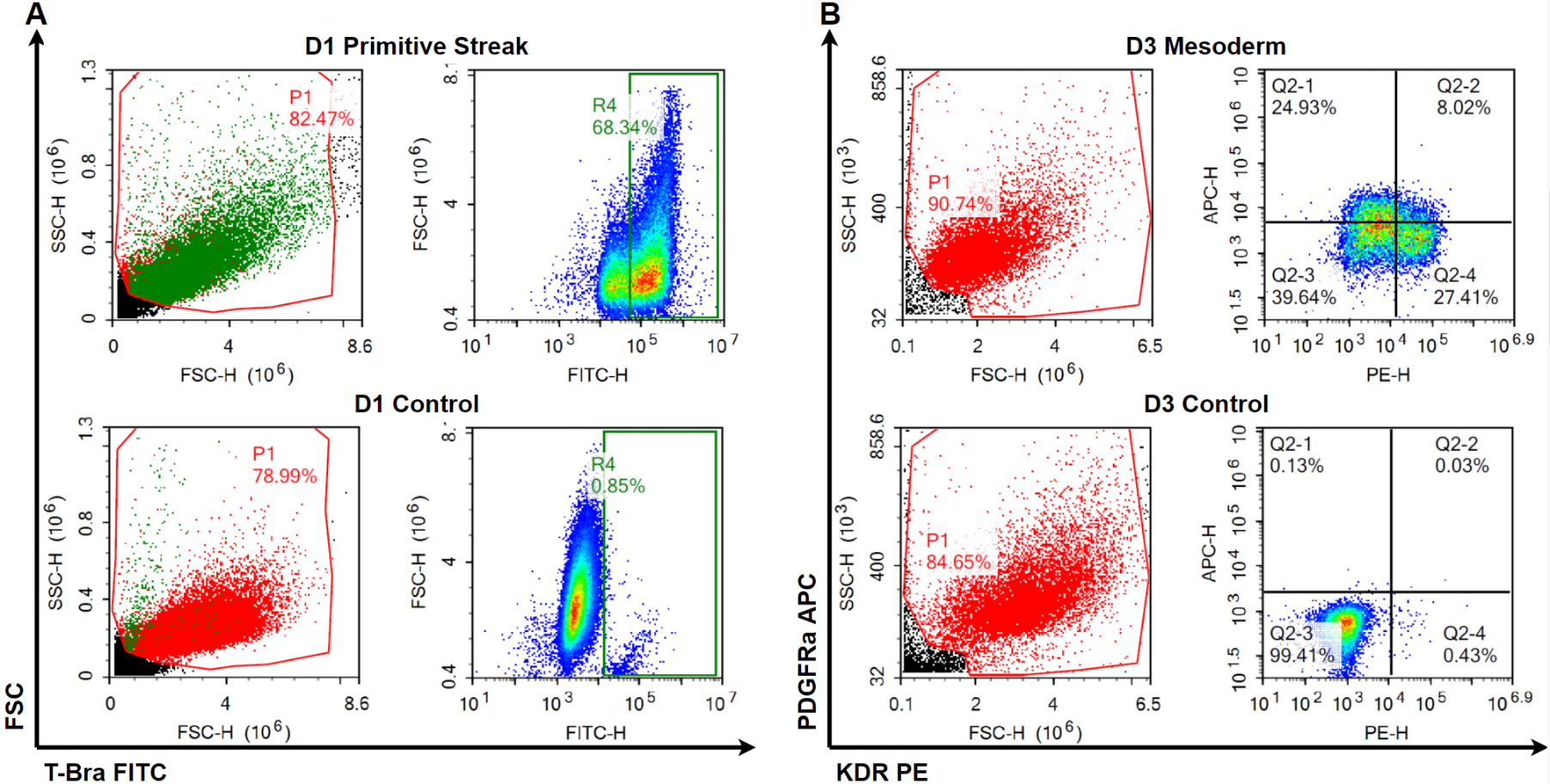


**Figure S6.**


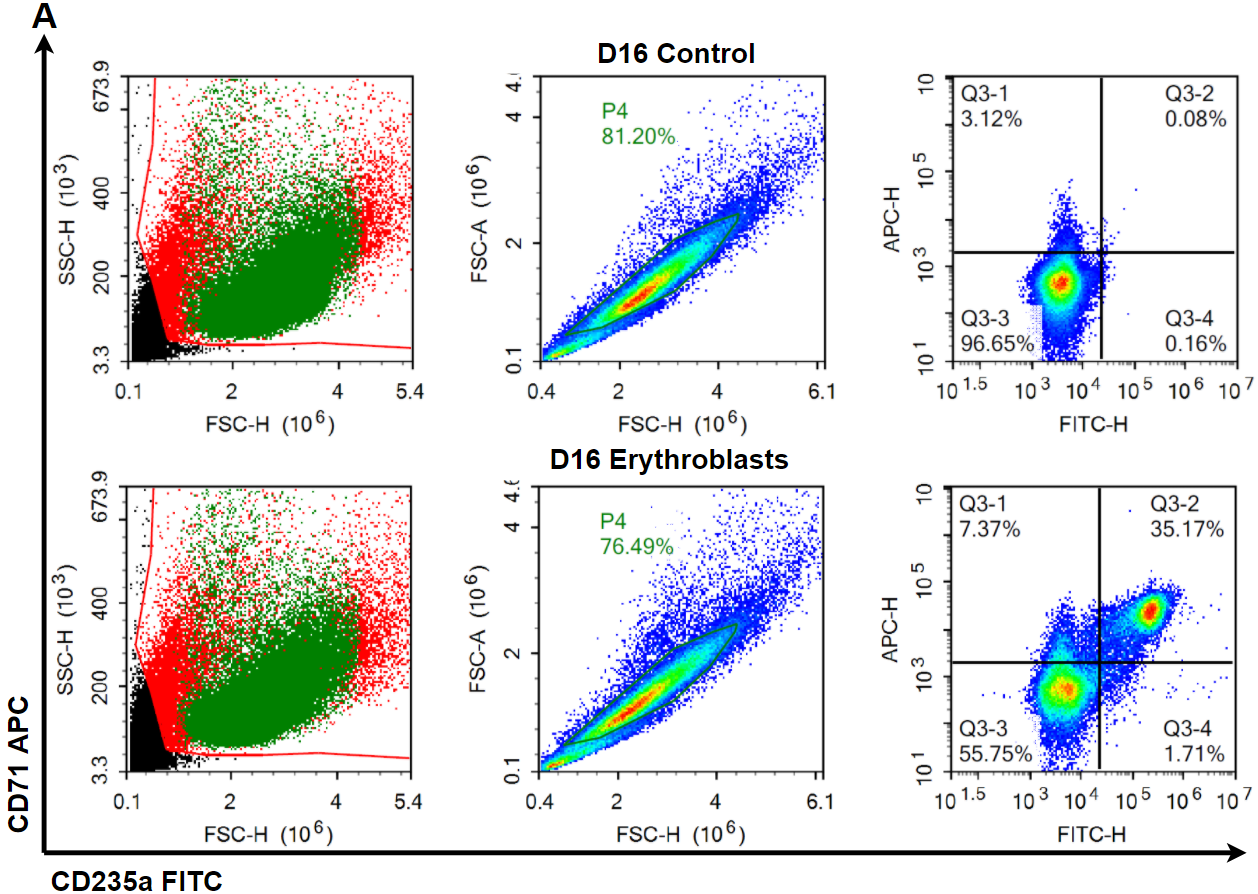

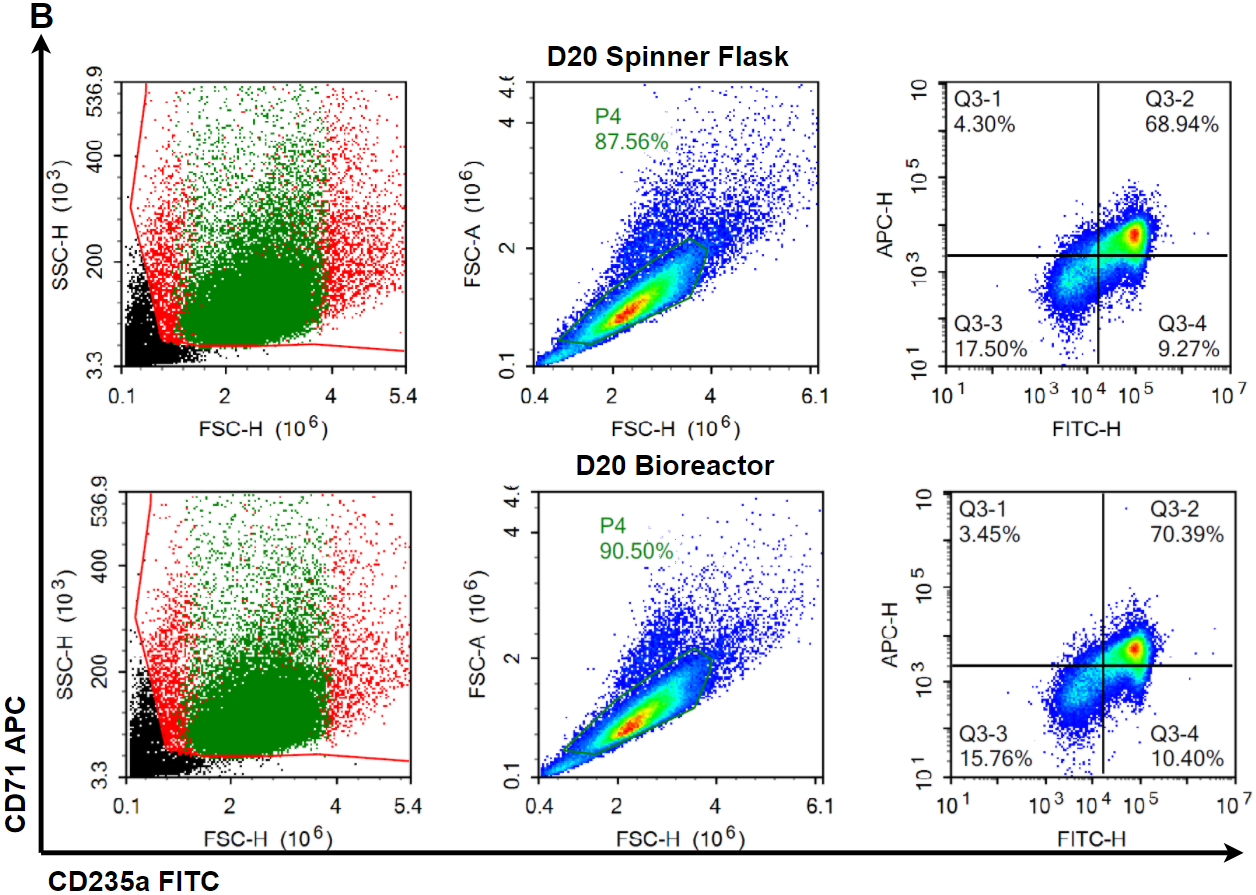


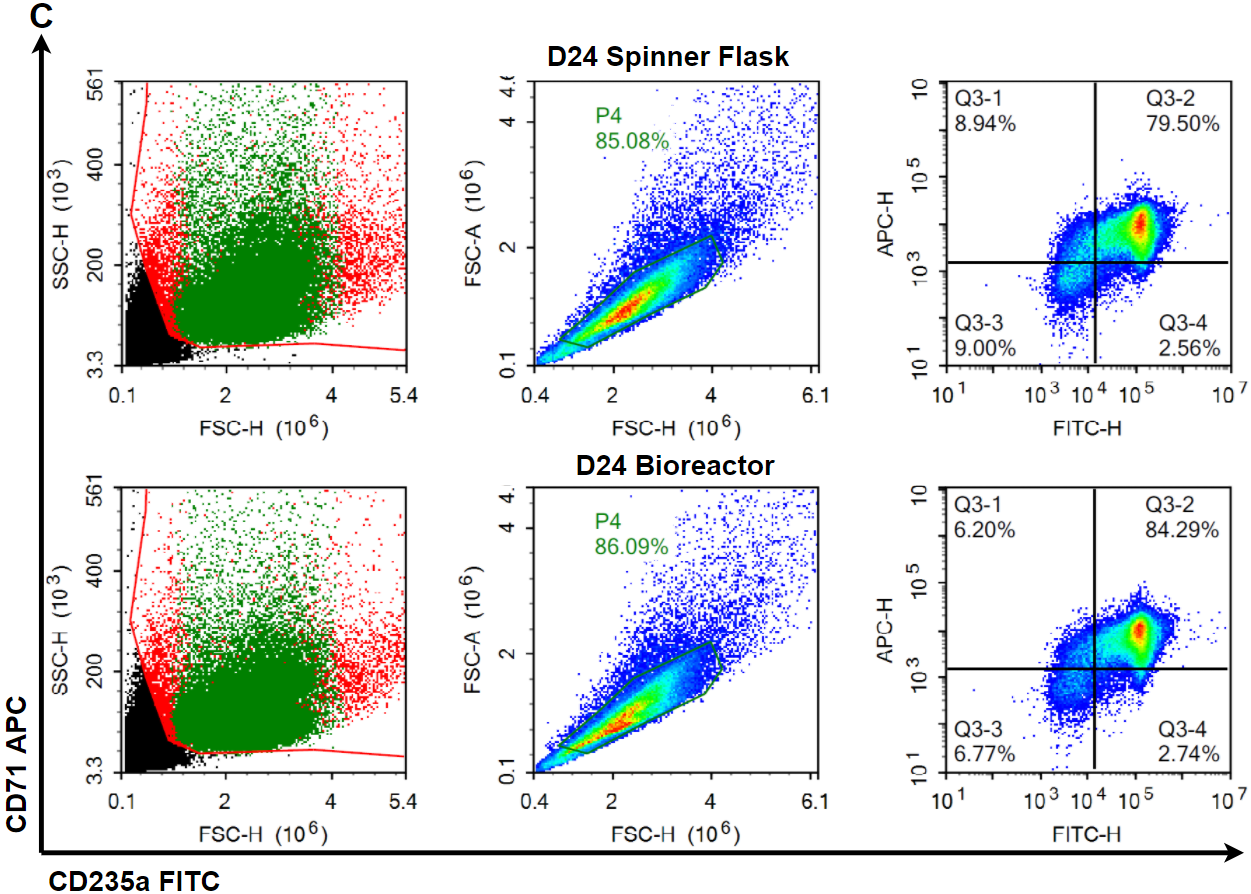


**Figure S7.**

References

The following references appear in the supplemental information:

1. Sivalingam, J.; Su, E.Y.; Lim, Z.R.; Lam, A.T.L.; Lee, A.P.; Lim, H.L.; Chen, H.Y.; Tan, H.K.; Warrier, T.; Hang, J.W.; et al. A Scalable Suspension Platform for Generating High-Density Cultures of Universal Red Blood Cells from Human Induced Pluripotent Stem Cells. *Stem Cell Reports* **2021**, *16*, 182-197, doi:10.1016/j.stemcr.2020.11.008.
